# An Overview of the Current Approaches in Drug-Resistant Bacterial Removal Within Wastewaters: Can We Move Towards Nanomagnet-Porphyrin Hybrids for Antimicrobial Photodynamic Inactivation (aPDI)

**DOI:** 10.1007/s00284-025-04222-0

**Published:** 2025-04-18

**Authors:** Mbalenhle Kabelo Nhlabathi-Chidi, Neo Mokgadi Mametja, Thabo Thokozani Innocent Nkambule, Usisipho Feleni, Tracy Masebe, Muthumuni Managa

**Affiliations:** 1 Institute for Nanotechnology and Water Sustainability (iNanoWS), Florida Campus, College of Science, Engineering and Technology, University of South Africa, Johannesburg, 1710 South Africa; 2 Department of Life and Consumer Sciences, College of Agriculture and Environmental Sciences (CAES), University of South Africa, Johannesburg, 1710 Florida South Africa

## Abstract

The rise in the occurrence of drug-resistant bacteria within wastewater treatment plants (WWTPs) and their dissemination into the ecosystem from the same WWTPs has created a prevalent crisis affecting the integrity of human life and water sources worldwide. Antimicrobial Photodynamic Inactivation (aPDI) can be explored in an effort to address this crisis and preserve natures integrity as it can incorporate environmentally sustainable and cost-effective disinfection strategies within wastewater treatment plants. aPDI is a technique introduced as a strategic approach to inactivate harmful Drug-Resistant Bacteria (DRB) that are ineffectively removed with current wastewater treatment strategies. The incorporation of Nanomagnet-Porphyrin Hybrid (NMPH) based aPDI illustrates notable microbial inactivation and innovatively introduces prospects of achieving affordable and ecologically beneficial disinfection within wastewaters since they can be recycled and reused. Furthermore the added advantage of NMPHs based aPDI lies in the generation of a high quantum yield of cytotoxic ^1^O_2_ due to a strong visible absorption ascribed to π–π* electronic transitions within the porphyrins. These properties are largely ascribed to the high coefficient of light absorption in a broad wavelength range allowing them to generate reactive oxygen species through a spin-forbidden intersystem crossing mechanism allowing them to demonstrate express disinfection of harmful pathogens. This review addresses the high inactivation profiles of NMPH based aPDI, its low operating costs and reusability as the potential of establishing NMPH based aPDI in nanotechnology wastewater remediation and microbial disinfection applications. The authors believe that this systematic review can stimulate new researchers and assist in the future development of this important field of research, especially when it comes to the aquatic environment and natural water resources and given the adequate attention this method can aid globally but more so within emerging economies to ensure potable water is delivered to all people.

## Introduction

Water contributes greatly to various industries and processes that pertain to life and its sustenance, and thus protecting and preserving this resource is deemed paramount globally and forms one of the six themes of the Eighth Phase of the International Hydrological Program [1, 2]. Increasing demands for water have elevated exertion of this already limited resource where some contributions to this problem include population growth and economic development which are mainly driven by increased trends of globalization within the developed world and emerging economies [1, 3, 4]. Thus, the urgency to develop refined strategies to provide potable water by improving water remediation disinfection practices becomes even more crucial due to cumulative acquired drug-resistance in bacteria and the resistant genes that they can transmit via various routes of horizontal gene transfer [5, 6]. The wastewater treatment sector has become challenged with the need to develop and introduce innovative protocols to disinfect and achieve complete removal of DRB and Antibiotic Resistant Genes (ARGs) as contaminants within wastewater which are currently ineffectively treated with existing WWTTs [7].

The emergence of DRB can be linked to wastewater that comes from hospital sources as these include additional stressors like biocides and heavy metals that have been linked to accelerated mutation rates and horizontal gene transfer (HGT) in bacterial populations as well as bacterial pathogens previously exposed to pharmaceutical agents thus causing selective pressure build-up leading to the transfer and acquisition of antibiotic resistant genes among bacteria [8, 9].

Researchers across the globe are now looking to address this crisis through exploration of aPDI utilizing nanomagnet-porphyrin hybrids (NMPHs) as immobilised insoluble supports that will harnesses natural sunlight to address ineffective antimicrobial treatment through a modified approach to solar water disinfection (SODIS) [10]. Insoluble immobilised porphyrin nanoconjugates when used in wastewater treatments of microorganisms further provide an environmentally friendly approach thought to have little effect on the environment and public health as no hazardous by-products are produced but also economical and ecologically friendly since it allows the PS to be extracted environmental fluids through application of an external magnet and reused from after treatment [11].

**Fig. 1:**
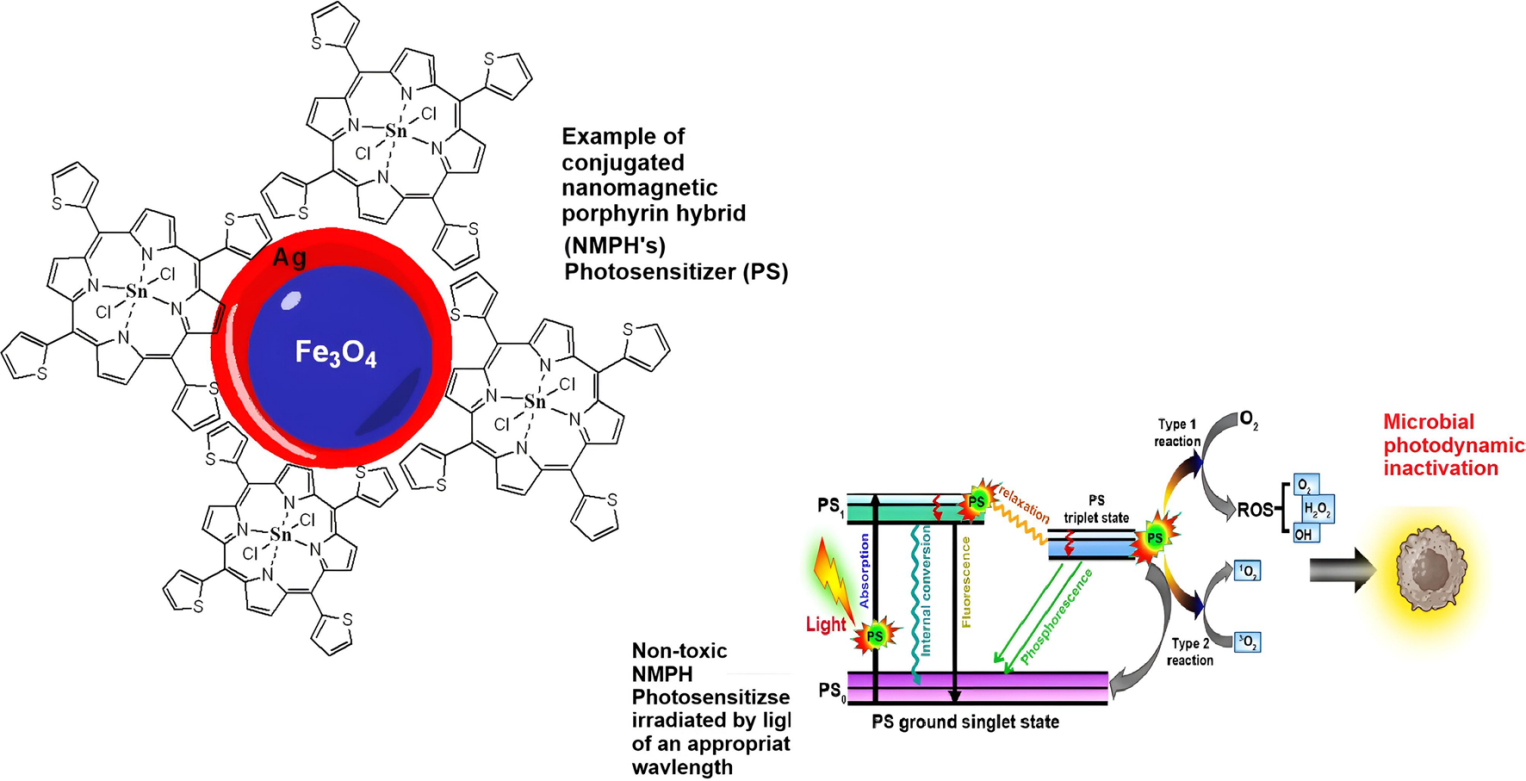
Diagram of truncated Jablonski diagram with an insoluble immobilised NMPHs PS and generation of singlet oxygen for effective use of aPDI in wastewater treatment plants. (Adapted with minor reconstruction with permission form from Ref Pallavi et al. 2024 [16]. (Copyright © The Author(s) 2024)

This approach aims to introduce an innovative perspective in the ways wastewater treatment can be approached especially regarding the removal of DRB (Fig. 1) and relies on the exploitation of a chemically stable photosensitizer such as a porphyrin, fabricated to enter microbial cells and inactivate them through oxidative stress upon the generation and release of cytotoxic ROS [12]. The aPDI approach takes advantage of the Singlet Oxygen (^1^O_2_) form of ROS harnessed from type II photochemical reactions during porphyrin photosensitizer (PS) irradiation.

For aPDI a photosensitizer is introduced to the bacterial solution, followed by irradiation. The PS is photoexcited from its ground state (S_0_) to its singlet excited state (*S_1_) when the PS-bacterial suspension interacts with light of a wavelength that is suitable for causing excitation at the λ_max_ of the PS spectral profile, the PS in the *S_1_ electronic transitory stage goes through divergent processes that could either favourably or unfavourably affect the production of ROS [13]. ROS production can occur when the PS in the *S_1_ state crosses across to an excited triplet state (*T_3_). Type I and Type II reactions are two possible coexisting mechanisms for ROS formation that the PS in the *T_3_ may go through. In a type I reaction, an electron (e−) or a proton (H+) is directly transferred from the PS in the *T_3_ to the cell’s substrates (such as the water in the bacterial cell membrane) to produce radical ions. These ions then react with oxygen to produce cytotoxic ROS, usually superoxide (O_2_–) and hydroxyl radicals (OH–) [14]. These species cause severe oxidative damage because they are extremely reactive and easily and quickly enter the bacterial cell [15]. Type II process involves the transfer of energy from the PS in the *T_3_ to the triplet ground state molecular oxygen (^3^O_2_) resulting in the generation of ^1^O_2_. Singlet oxygen is the molecular oxygen in its electronically excited state and is less stable; moreover, it is the predominant cytotoxic substrate amongst all the ROS in aPDI. ^1^O_2_ interacts with various components within the cell such as proteins and the deoxyribonucleic acid (DNA) bases [13, 15].

This review focuses on the advantages of insoluble immobilised porphyrin centered aPDI and the ways in which this approach can offer cost-effective and complete sustainable disinfection of DRB within wastewater treatment plants, supplementing current technologies and bridging the gaps of their limitations surrounding the inability to adequately address drug-resistant bacteria whilst introducing a renewed innovative protocol to wastewater treatment processes that will aid in affording delivery of potable water and addressing the crisis of water scarcity.

## Limitations Surrounding Current wastewater Treatment Technologies in Removal of Drug-Resistant Microbes

Various WWTTs are utilized in the present day, and vary widely, being either biological, physical, or chemical in nature and in some instances an overlap of two or more technologies unfortunately still, the robust nature of microbial species and their strong adherence to surfaces enable the drug-resistant pathogens to circumvent these strategies. Highlighting on the drawbacks with existing practices supports the importance of needing to introduce aPDI in wastewater treatment plant disinfection protocols [17, 18].

Consider membrane-based separation technologies, these constructs offer great durability and cost-effectiveness, with decreased sludge generation [19]. Mosqueda-Jimenez and co-workers were able to show how these technologies offered flexible operation, and high nitrification performance to facilitate great retention of more than 5 log units of bacteria [20].

**Fig. 2:**
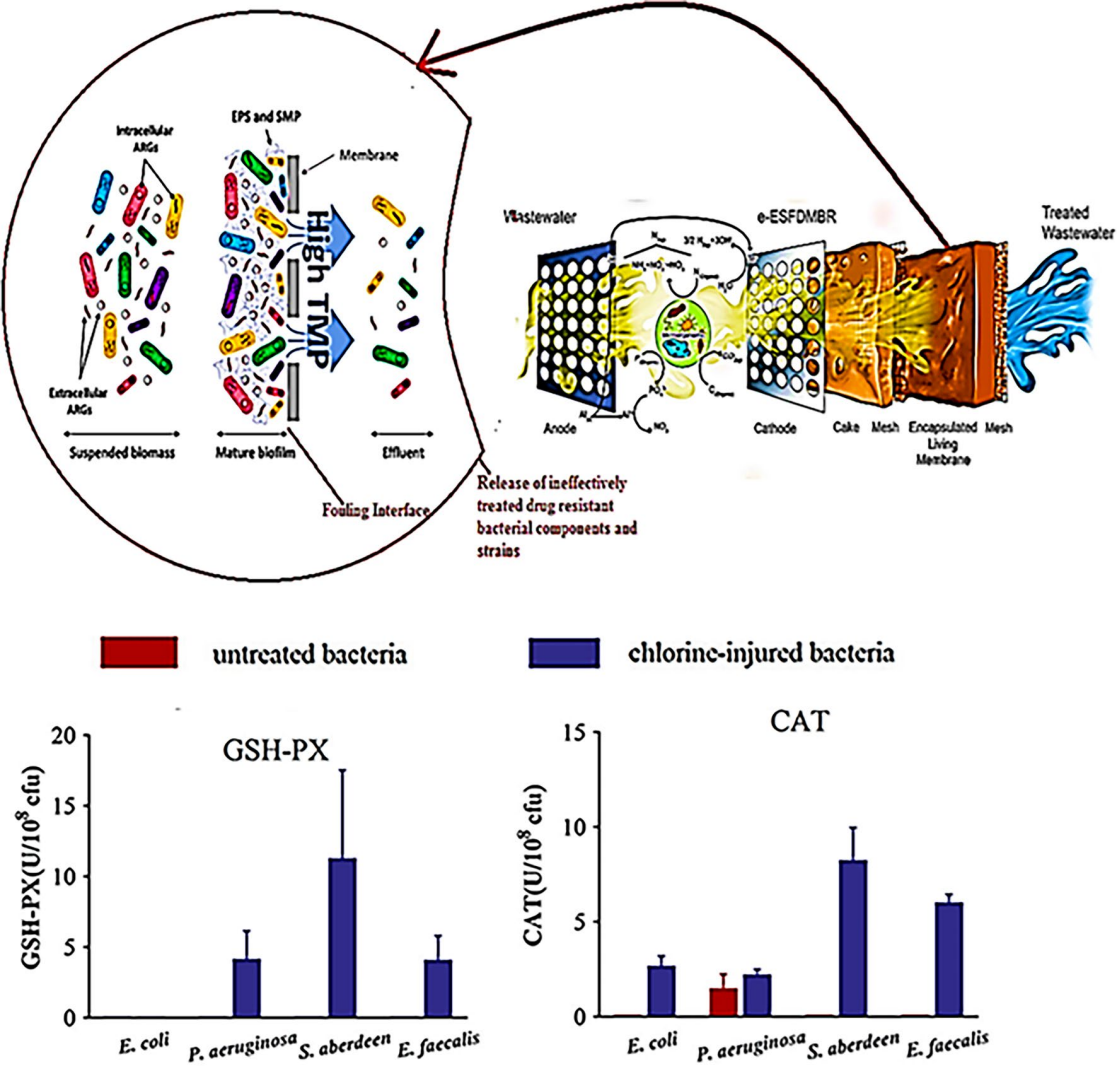
Illustration of (**a**). The route of fouling and the subsequent impact of release of DRB from the effluent due to antimicrobial disinfection failure as a result of fouling and (**b**). The chlorine resistance profiles of bacteria shown through increased activities of CAT and GSH-PX enzymes within the bacterial entities. (Adapted with permission from Ref Millanar-Marfa et al. 2022 [26]. Copyright ©2022 Springer Nature and Ref Jin et al. 2020 [33]. Zarei-Baygi et al. 2019 [23] Copyright ©2020 American Chemical Society

However membrane-based separation technologies are limited in that they are susceptible to fouling [21, 22]. Absolute abundance decline patterns of ARGs in the suspended biomass during influent treatment operations were reported by Ali Zarei-Baygi and colleagues’ highlighting the discovery of significantly greater colony-forming units (CFU) in fouling layers which subsequently normalized abundances of antibiotic resistant components, further promoting bacterial adsorption onto the membrane, thus increasing the possible opportunities for horizontal gene transfer of resistant genes across bacterial entities as shown in (Fig. 2) [23, 24].

Even among elaborate membrane constructs such as electro-encapsulated self-forming dynamic membrane bioreactors, that couple electric field applications to remove organic residues and mineral salts from wastewater and control fouling, it has being revealed that no substantial account regarding the removal efficiency of DRB is reported [25, 26]. Treated effluent data showed that total ARG abundance in the effluent of the membrane construct was unaffected by the degree of fouling and that the highest absolute abundance of antibiotic resistant entities was concurrently present in the fouled membrane effluents [26].

Another WWTT widely used for bacterial disinfection is the chlorination protocol familiarized by its ability to generate hydrochloric acid and hypochlorous acid when dissolved in water [27]. The chorine will interact with bacterial entities altering their structural compositions, and in so doing distorting the performance capacity of the bacterial enzymes, and thus suppressing the foundation of bacterial nutritional processes, preventing their growth and rendering them inactive [28, 29]. Unfortunately, due to increasing acquired resistance towards chlorination being expressed among various forms of bacteria this disinfection technique has also once again been proven insufficient at achieving total microbial eradication with pronounced antimicrobial efficacy [30, 31].

M. Jin and co-workers demonstrated how the chlorination process encouraged the horizontal transfer of plasmids through natural transformation, leading to the transfer of chlorine-injured adept pathogen from non-DRB to DRB and the exchange of ARGs between bacterial families [32]. Their findings revealed that diverse resistance to sodium hypochlorite was expressed by *Salmonella aberdeen, Pseudomonas aeruginosa, Escherichia coli,* and *Enterococcus faecalis* with transferable RP4 additionally being routinely released from killed susceptible donors [33].

A biologically competent cell could be transferred by RP4 with an improved transformation frequency of up to 550 times when compared to the associated unresolved bacteria, as evidenced by the survival of chlorine-tolerant injured bacteria with enhanced cell membrane permeabilization and a strong oxidative stress response. Following chlorination, Catalase enzyme (CAT) was expressed by all four studied bacterial models, with *S. aberdeen* exhibiting the highest CAT activity of 8.3 U/10^8^ CFU. Once more subsequent to chlorination after RP4 exposure the bacteria which previously showed negligible GSH-Px) activity, showed enhanced GSH-Px activity of up to 11.3 U/10^8^ CFU as show in (Fig. 2). All these findings suggest that the NaClO exposed bacteria developed robust oxidative stress response [33].

Such limitations with current WWTTs have propagated the emergence and prevalence of DRB. A circumstance now presenting great concern to public health and as such further highlights the urgent need to develop suitable porphyrins to incorporate strategies such as aPDI for wastewater microbial disinfection.

## Advanced Oxidation Principles Used in Wastewater Treatment Methods that Support Introduction and Incorporation of aPDI

The existing limitations surrounding DRB removal efficacy with current wastewater treatment strategies, emphasize how imperative it is to establish alternative methods for improved disinfection of DRB within wastewater treatment systems. The aPDI technique has been widely explored on the bench scale and gaining vast and rapid momentum globally among researchers as a novel strategy to be introduced into the wastewater treatment sector due to its proven performance in the total removal efficacy of DRB by photo inactivation [34–36]. aPDI draws on similar bacterial inactivation mechanisms as the numerous forms of Advanced Oxidation Processes (AOPs) described in (Table 1), which are already implemented within wastewater treatment plants and boost superior efficacy in total eradication of DRB.

**Table 1:** Summary of the main forms of Advanced Oxidation Process Techniques and the generation of the respective ROS applicable for microbial inactivation in wastewater

Advantages of oxidation technique	Modes of Applicable Inactivation Mechanisms	References
Radiation	Utilization of two radioactive sources, the electron beam irradiation carried out under an electron accelerator and gamma irradiation performed using a cobalt 60 or cesium-137 source. H_2_O_2_ and O_3_ radical accelerates inactivation	[43]
Photocatalysis	Utilization of high energy associated with UV radiation of a wavelength shorter than 190 nm yield powerful oxidizing species	[55]
Cavitation (sonolysis)	Formation of hydroxy radicals under ultrasound (US) irradiation processes in the presence of different gases as well as combination with other processes (US/O_3_, US/H_2_O_2_)	[55–57]
Electrochemical oxidation technologies	Involvement of an anode, where water molecules are oxidized leading to the formation of radicals, hydrogen gas	[54, 57]
Fenton based reactions	Generation of hydroxy radicals in acidic media via under a number of cycles which incorporate ferrous or ferric ion-based catalysts	[56]
Ozone based processes	Water ozonation involves hydroxy radicals that are generated via the SBH model and serve as a promising treatment of recalcitrant organic compounds such as organic pesticides and pharmaceuticals in wastewater	[43]

Before the introduction and application of AOP related treatment methods, the intricate resistance patterns of bacteria posed grievous challenges for the scientific community, especially with regard to wastewater, particularly hospital effluent disinfections [37, 38]. This was attributed to the porins and efflux channels which contribute to antibiotics resistance and the varying abilities of different bacteria to withstand, heat, ultra-violet (UV) and chemical disinfectants associated with conventional wastewater treatment technologies [39, 40]. The introduction and implementation of AOP based technologies produced notable inactivation and elimination of resistant bacterial strains such as *A. baumannii* strains from wastewater.

The two technologies AOPs and aPDI share similarities in that both incorporate the use of reactive oxygen species to incite cytotoxic oxidative stress to inactivate bacteria and so the introduction of porphyrin mediated aPDI as a supplementary protocol can offer a welcomed transition as it debuts as an adapted approach to AOP which has proven performance efficacy in achieving total inactivation of DRB within WWTPs [41–44]. Some of the most noteworthy accounts in the performance of AOPs with regards to the removal of recalcitrant bacteria from wastewater is seen in work by Pranjal and Co-workers.

**Fig. 3:**
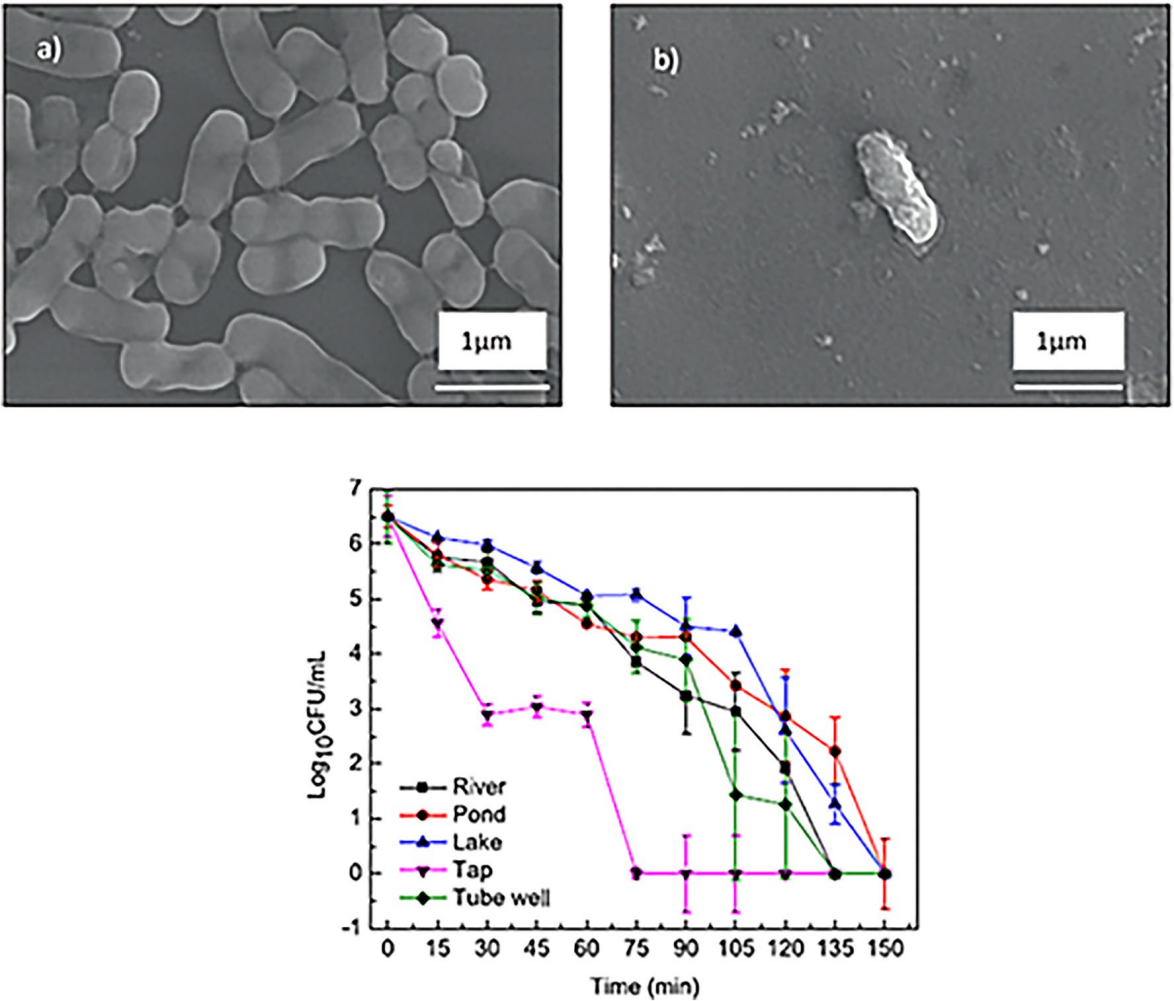
*A. baumannii* **a** before and **b** after a 90-min SF treatment as seen through scanning electron microscopy (SEM) and **c** the impact of SF treatment on various real water samples collected from a river, pond, lake, tube well, and tap water. (Ultrasound frequency: 40 kHz, bacterial loading: 5 × 106 CFU/mL). (Adapted with permission from Ref Pranjal et al. 2023 [46]. Copyright ©2023 Elsevier B.V. All rights reserved)

The researchers explored the inactivation of environmental *A. baumannii* with ultrasound assisted Fenton (Sono-Fenton: SF) based reactions. A nosocomial pathogen responsible for causing bacterial meningitis, urinary tract infections, surgical wound infections and pneumonia with significant ability to develop resistance to most of the conventional antibiotics [45, 97]. Their key findings revealed inactivation of ≈ 5 × 10^6^ CFU.mL^−1^ of *A. baumannii* from various real water samples after only 90 min of SF treatment under weak acidic conditions using 20 mg L^−1^ of H_2_O_2_ and 2 mg L^−1^ of Fe^2+^ to yield more than a 99% inactivation efficiency with no reactivation of the bacteria for 96 h (Fig. 3a–c) [46–48]. They substantiated long-term bactericidal effects against *A. baumannii* through ROS that resulted in residual stress of the bacteria [46].

Similar observations of bacterial activations were made by Eghbali and co-workers [49]. The functional aspects of AOPs are centered around the in-situ productions of high ROS mostly the hydroxyl radicals for wastewater treatment applications to eliminate water pathogens and pathogenic indicators [50]. This is largely attributed to the highly reactive and nonselective nature of the hydroxy radical, as well as its high standard potentials of 2.8 V against the 1.55 V of the normal hydrogen electrode (NHE) in acidic and basic environments [51, 52]. This high voltaic difference increases its oxidizing performance enabling it to breakdown a variety of dangerous pathogens and harmful chemicals to CO_2_ and inorganic ions [53, 54].

The express success illustrated with AOPs in overcoming disinfection of DRB in wastewater treatment, strongly promotes the need for introduction and implementation of NMPHs mediated aPDI based methods as a reformed protocol for wastewater antimicrobial treatment [58].

## Nanomagnet-Porphyrin Hybrids in aPDI as Potential Wastewater Disinfectants

**Fig. 4:**
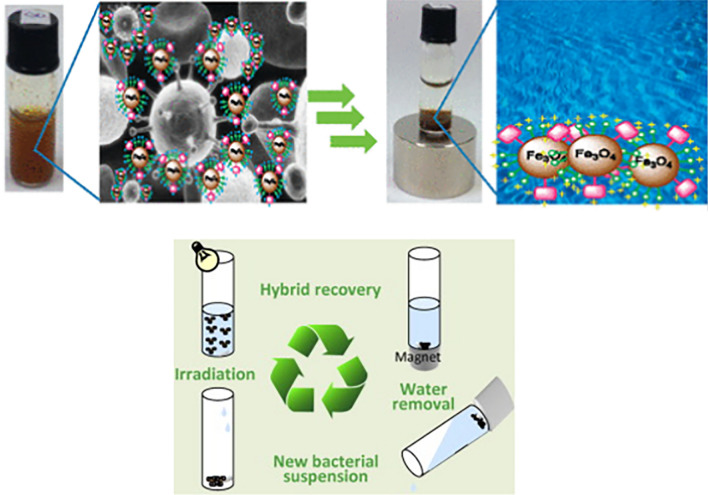
Illustration of use and recovery of nanomagnetic hybrid photosensitizers systems and potential antimicrobial applications for wastewater treatment. (Adapted with permission from Ref Alves et al. 2014 [59]. Copyright ©2014 Elsevier B.V. All rights reserved and Ref Carvalho et al. 2010 [62] Copyright ©2010 American Chemical Society All rights reserved)

Porphyrins immobilised onto inert magnetic nanoparticles (MNPs) solid supports as photosensitizers in aPDI are thought to provide an affordable and ecologically beneficial wastewater disinfection method because they can be recycled and reused [59]. Numerous successful lab-scale aPDI inactivation trials on wastewater samples with NMPHs against World Health Organization (WHO) high priority drug-resistant bacteria have been reported in recent years. Work conducted by Carvalho and co-workers explored the disinfection of T4-like Bacteriophage with novel multicharged nanomagnet-porphyrin hybrids and the recoverability and reusability of the nano construct (Fig. 4) [60–62]. The research group demonstrated how well these novel multicharged nanomagnet-porphyrin hybrids photo inactivate bacteria and phages whilst still maintaining their stability in water and post photoinactivation treatment. These materials are innovative photosensitizers for disinfecting wastewater because of their exceptional antibacterial activity and ease of recovery when an external magnetic field is applied [63].

Alves and co-workers conducted a study surrounding the recovery and reuse of NMPHs for wastewater disinfection. The researchers studied the photoinactivation of *Allivibrio fischeri* and recyclable capacity of NMPHs namely 5-(pentafluorophenyl)-10,15,20-tris(1-methylpyridinium-4-yl)porphyrin-tri-iodide] coupled to cationized silica-coated magnetic nanoparticles of Fe_3_O_4_ and CoFe_2_O_4_ respectively_._ Both hybrids showed to be photostable whilst maintaining their magnetic characteristics during the photoinactivation studies conducted at irradiance of 40Wm^−2^ and a total light exposure of 345 Jcm^−2^) [59]. The overall values of bacterial inactivation after a six-cycle reuse were ~ 42 log10 colony-forming units (CFU) mL^−1^ with Fe_3_O_4_-porphyrin hybrid in 21.5 h and ~ 38 log10 CFU mL^−1^ with CoFe_2_O_4_-porphyrin hybrid in 27 h. Their study emphasized the exceptional antibacterial qualities and photostability of NMPHs, as well as their potential for recycling and reuse as a promising wastewater disinfection protocol.

Magnetic nanoparticles (MNPs) are a highly beneficial class of nanoparticles for porphyrin immobilisation for aPDI water disinfection applications. Typically, they are coated with a non-magnetic, relatively inert substance to maintain their magnetic properties, or they can be embellished with another inert substance, like silver or an amorphous silica shell coat, which can shield against oxidation of the metallic core and make it simple to immobilize additional active sites [60]. Some important attributes which include easy substitutions with a large variety of nucleophiles, stability in aqueous media, hydrophilicity achieved through derivatization are key aspect consider in the synthesis of porphyrins developed for hybrid MNP immobilisation and aPDI. Also, key is the preparation of suitable physical, electronic and optical properties for water disinfection applications. Table 2 provides an outline of some various NMPHs developed for microbial photoinactivation.

**Table 2:** Outline of nanomagnet-porphyrin hybrid nanostructures developed for photoinactivation of a range microorganisms or pollutant degradation

Nanomagnet-porphyrin hybrid	Photoinactivated Bacterial model/Photo degraded pollutant	References
	*Allivibrio fischeri* (gram(−))	[59]
	17β-estradiol	[60]
	*Staphylococcus aureus* (gram(+))	[61]
	*Staphylococcus aureus* (gram(+))	[61]
	*Escherichia coli* (gram(−)) *Enterococcus faecalis* (gram(+)) T4-like phage (bacterial phage)	[62]
	*Staphylococcus aureus* (gram(+))	[63]
	*Staphylococcus aureus* (gram(+))	[63]

The added advantage of NMPHs based aPDI lies in the generation of a high quantum yield of cytotoxic ^1^O_2_ due to a strong visible absorption ascribed to π–π* electronic transitions within the porphyrins [64–66].

These properties are largely ascribed to the high coefficient of light absorption in a broad wavelength range allowing them to generate reactive oxygen species through a spin-forbidden intersystem crossing mechanism [67, 68]. Previous studies on synthetic porphyrins concentrated on highlighting their photochemical activity, which enables their use in photodynamic inactivation applications on microbes [69, 70].

Sufficient modifications through immobilisation of the porphyrin photosensitizer structure promotes a higher generated yield of these highly reactive species with increased stability for water disinfection [71, 72]. The efficacy of ROS in bacterial inactivation applications lies in their spin-multiplicity which matches the multiplicity of the ground state of a number of molecules allowing ^1^O_2_ to perform as an effective oxidant usually attacking the carbon double bonds in organic molecules such as lipids, amino acids, proteins, and nucleic acids [73, 74].

The incorporation of solid supports further attenuates the existing properties of porphyrins and have been proven to exhibit enhanced antimicrobial properties [75]. This is achieved by optimising the porphyrin structures against agglomeration-related coalescence and deactivation, limited reusability and recovery [76]. This allows the effects of non-covalent interactive and hydrophobic forces, hydrogen bonding, and aromatic stacking that contribute to coalescence related to agglomeration to be avoided [77].

Such phenomena are encouraged by self-assembly of porphyrins and results in nanostructures that are frequently well-defined or not [78]. Incorporation of nanoparticles to porphyrin structures afforded increased ^1^O_2_ yields resulting in higher photodynamic inactivation efficacy through the conversion of near-infrared light to visible light [65]. Developing new generations of metal supported porphyrin nanomaterials, as PSs improves photostability and encourages higher and more efficient singlet oxygen generation (SOG). This occurs because of acquired synergistic effects achieved through the combination of these metal supported porphyrin nanomaterials which contribute greatly in the reactive oxygen species generating process.

Superior photocatalytic materials are produced as a result of such contributions as pphotoexcited electrons can be stimulated and transported to the dissolved oxygen (DO) in the medium, where it can be abstracted by the metalloporphyrin, resulting in the generation of •OH radicals [79–81]. Then, as a result of irradiation absorption, ROS are produced, and these species have the ability to oxidize the contaminants present in wastewater [82]. Metal nanomaterial-based PS conjugations comprise of plasmonic coupling which improve the SOG efficiency of PSs and this can often be appreciated with silver and gold metal nanomaterial. Plasmonic coupling depends on the interactions of light with the nearby PS and plasmonic nanoparticles [83, 84]. This plasmon-enhancement technique can be used to speed up the SOG for many kinds of PS even porphyrin-based PSs and provide the added benefit of further enhancing the photostability of the PS whilst also reducing the susceptibility to photobleaching. The heterogenous porphyrinic materials further express increased photostability during phototreatment operations and a capacity to retain their high ROS generation [85, 86].

A consideration of work by Lin and co-workers fully highlights the vast optimization potential that nanomagnet-porphyrin hybrid heterogenous systems have in aPDI particularly when looking to utilise this technology in wastewater treatment to address DRB [87, 88].This group focussed on the antibacterial action Gallium Mesoporphyrin IX (GaPpIX) has as an aPDI agent as well as activities of GaPpIX, in complexion with haemoglobin (GaHb), and Ag nanoparticles (GaHb-AgNP) [89].

Manipulation of GaPpIX as a haem analogue as one possibility for additional research to promote favourable absorption by cell-surface hemin receptors (CSHRs), facilitated the direct acquisition of hemin along the bacterial surface [90, 91]. Their investigations revealed stimulated expression of CSHRs in cultivations of *S. aureus* in iron-limited conditions which facilitated a greater uptake of GaPpIX [92]. The iron-deficient condition is relevant because bacteria have developed tactics such as hemin harvesting to overcome iron deficiency, giving rise to opportunities for exploiting hemin uptake for pathogen-specific aPDI [90, 93].

Exploration of the aPDI effects revealed GaPpIX is an effective and fast-acting aPDI agent against laboratory strain of *S. aureus* (PC1203) and also clinical isolates of Methicillin-resistant *Staphylococcus aureus* (MRSA) [94]. The aPDI potency of GaPpIX was established using a light-emitting diode (LED) array with monochromatic emission at 405-nm; 1.40 J/cm2 to irradiate bacteria treated with GaPpIX, TMPyP, and Mesoporphyrin IX (PpIX) PpIX as reference PS [95–97]. The aPDI activity of GaPpIX against *S. aureus* is rapid, with antimicrobial action (> 99.9%) at 59 nM after 10 s of irradiation, a 2000-fold increase in potency relative to GaPpIX dark toxicity and total eradication of more than 6 log10 reduction was observed at 235 nM [98].

**Fig. 5:**
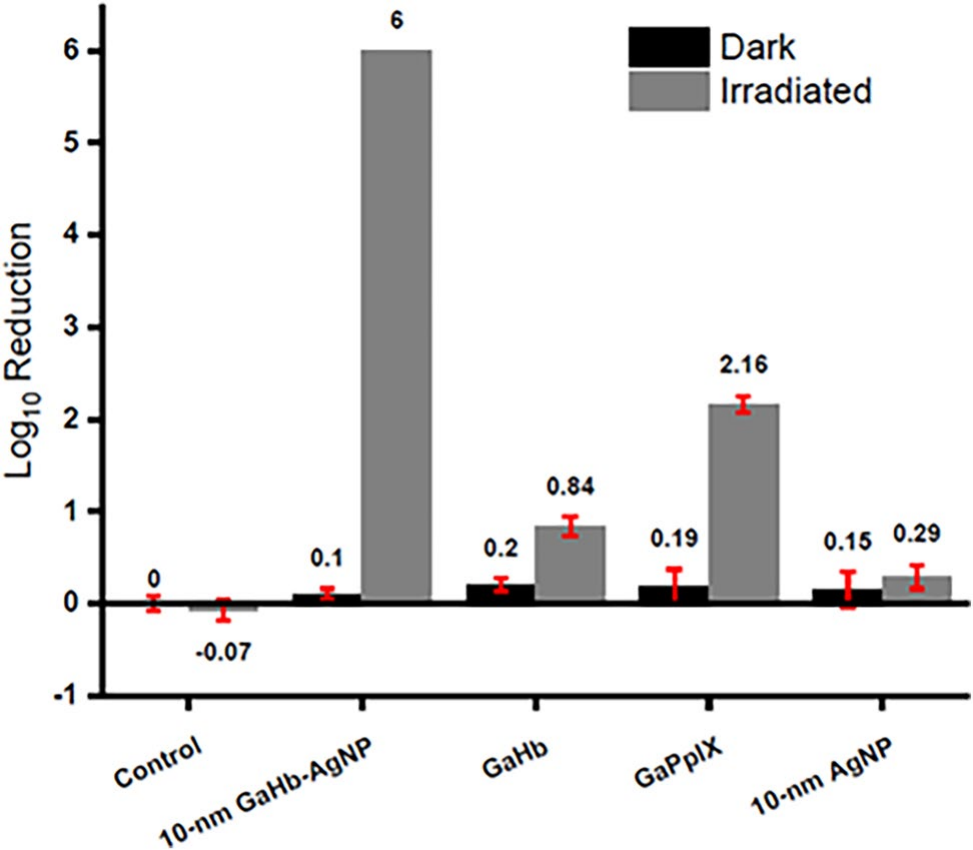
Log reduction in *S. aureus* cell viability when treated with PBS pH 7.4 (control), 10 nm GaHb-AgNP (5.7 μg/mL), GaHb (0.66 μg/mL), GaPpIX (27 ng/mL), and 10 nm AgNP (5 μg/mL), after a 10 s exposure to a 405 nm LED source (140 mW/cm^2^). (Adapted with permission from Ref Morales-De-Echegaray et al. 2019 [94]. Available in PMC 2019 November 09)

By comparison, the aPDI activity of TMPyP and PpIX were 8 and 32-fold less potent than that of GaPpIX and a similar potency also expressed against several clinical isolates of MRSA using the same condition [88, 96]. GaPpIX has poor solubility in water at physiological pH, but is highly soluble in organic solvents such as Dimethylsulfoxide (DMSO) which it as an ideal compound for aPDI applications in wastewater treatments [96]. When coupled with Silver Nanoparticles (AgNPs) it GaPpIX expressed increased ^1^O_2_ production by up to three orders of magnitude and resulted in highly efficient aPDI against both gram-positive and gram-negative bacteria shown in (Fig. 5) [97, 98].

This is owing to the strong resonant coupling between the surface plasmon and PS, meaning that light-absorbing PS near metal nanoparticles have a stronger absorption cross section due to the localized surface plasmon [99]. The strength of plasmon–PS resonance coupling is highly sensitive to the spectral overlap between the molecular absorption and the surface plasmon bands of metal nanostructures [100, 101].

The resonant coupling between the absorption band of J-aggregate molecules (a self-organizable supramolecular dye) and the plasmonic band of AgNPs greatly enhanced the exciton lifetime, excitation of GaPpIX is further amplified by AgNPs, further enhancing the production of ^1^O_2._ when considering the researchers application of GaHb-AgNPs in aPDI investigations [88, 94, 99, 102].

Great inspiration from work similar to that of Lin and co-workers can be drawn for the utilisation of supported i porphyrins conjugated to metal nanoparticles for applications of aPDI (Table 3) in wastewater treatment and migrate from the bench scale into pilots that can afford a highly effective approach to address drug-resistant bacteria within wastewater.

**Table 3:** The main advantages nanomagnet-porphyrin hybrid coupled systems for Antimicrobial Photodynamic Inactivation for wastewater treatment applications

ITEM	aPDI Advantage	References
1	Broad-spectrum of action: PSs efficiently inactivate bacteria, viruses, fungi, and parasites in both dormant and vegetative states contrary to chemotherapy	[95, 103]
2	Efficient phototoxic activity against both wild and drug-resistant microbial strains	[100, 104]
3	Lack of selection of photo-resistant microbial species, mutagenic potential is low	[103]
4	A high selectivity in the killing of pathogens as compared with existing wastewater techniques	[9, 32, 34, 37, 82]
5	A high selectivity in space and time; the microsecond short lifetime and high Reactivity of singlet oxygen	[32, 106]
6	Possibility to reuse a PS, making the technology less expensive and environmentally friendly	[58, 96, 105]

## The Key Benefits and Underlying Principal Mechanisms of Nanotechnology Applications Towards Wastewater Disinfection

The impressive antimicrobial performance profiles observed and highlighted with NMPHs centred aPDI affords insightful information surrounding the importance of nanotechnology applications in wastewater disinfection and the benefits that can be exploited with its use. Nanotechnology has shown significant promise in addressing several challenges around environmental sustainability as it pertains to wastewater disinfection whilst introducing constructs that can be uniquely fabricated in different forms as antimicrobial agents to decontaminate water as described using NMPHs as examples [107, 108].

Engineering nanomaterials for wastewater disinfection applications offers special qualities above their bulk counterparts, including high reactivity, customized architectures, and customizable surface characteristics. These constructs offer remarkable catalytic, adsorptive, and optical qualities which are very crucial components for effective water disinfection. Incorporating nanoscale metal oxides such as iron as observed with NMPHs allows an added benefit of increased binding mechanisms and microbial activity [108–110].

The acceptability of nanotechnology for wastewater microbial disinfection is ultimately influenced by its effectiveness and reduced costs. This is particularly appreciated in nanotechnology applications within emerging economies which typically addresses the most basic disinfection requirements [111]. Nanoparticles of lower purity can be fabricated to increase the viability of employing them to clean wastewater. Such examples are reported by Pan and co-workers where fabrication of amino-fullerene photocatalysts was reported that included the substitution of ultrapure C_60_ with fullerene soot resulting in a 90% cost decrease with negligible loss in contaminant removal efficacy and sustained long-term reusability. A very important attribute as fullerenes have been proven in numerous accounts in literature to facilitate effective antimicrobial photodynamic inactivation over broad classes of microbial cells. Impressive inactivation profiles were confirmed on *S. aureus* by Mroz and co-workers wherein a broadband-pass filter light within the visible spectrum λ_(400–700 nm)_ was used to excite the fullerenes and inactivate *S. aureus* [112, 113].

Further reports of the benefits in cost reduction of nanotechnology wastewater disinfection applications include magnetically separable multifunctional nanomagnetic constructs that allow for numerous reuse cycles and photocatalysts that maintain their activity thus promoting improved water disinfection protocols with reduced costs a fundamental key benefit that can be explored with nanotechnology applications such as those that involve NMPHs based aPDI for wastewater disinfection [113].

Fernández and co-workers demonstrated the effective aPDI treatment on recombinant bioluminescent *E. coli* and reusability using 5,10,15,20-tetrakis(pentafluorophenyl)porphyrin immobilised onto amorphous silica shell coated magnetite nanoparticles. The group reported the use of magnetic nanoparticles (MNPs) to enable fast, easy and inexpensive recovery of NMPHs from the aqueous media by applying a magnetic field, and their consequent reuse [113, 114]. Following inactivation of light irradiation over 270 min with a fluence rate of 4 mW·m^−2^, bacterial suspensions revealed log reductions averaging 7.9 ± 0.3 over 5 recycling runs, in which photosensitization activity was retained with negligible loss of PDI efficiency. After 5 runs the group reported only a fractional decrease in log reduction of 1.9 log which was accounted to the marginal loss of the NMPH complex during recycling treatments [114].

Some of the primary mechanisms of disinfection observed with NMPH nano constructs include exploitation of enhanced microbial binding affinities often expressed with magnetic engineered nano constructs such as conjugated ferric oxide (Fe_3_O_4_) magnetic NPs. These constructs were shown to exhibit superior removal efficiency of harmful pathogens and wastewater contaminants as illustrated in work by Sadak and co-workers [115]. NMPHs comprising Ferric oxide (Fe_3_O_4_) magnetic NPs (MNPs) can be conjugated to polyacrylic acids and further functionalized with dyes such as CR azo dye or porphyrin moieties [114]. Upon irradiation such constructs express profound removal efficiencies of wastewater contaminates by photocatalytic processes found to be maximal at 6.5 pH and 45 min of reaction time the kinetics of which profiled to best correlate with pseudo second-order model [109, 114]. An alternative mechanism of disinfection related to magnetic nano constructs in wastewater applications includes cation exchange over immobilized nanoscale zero valent iron constructs. This involves elevated bacterial removal rates caused by chemical reduction in which Fe^0^ and Fe^2+^ solid materials remove microbial contaminants for water disinfection [116].

Other key mechanisms of nanotechnology-based constructs for water disinfection include bandgap reduction for photocatalytic antimicrobial reduction against pathogenic contaminants often with nano constructs involving Zinc and Titanium oxides [117]. Bandgap reduction can be achieved by anion doping at interstitial and substitutional sites of the nanoparticle lattice frameworks. This instigates a photocatalytic redox reaction within the visible light absorption spectrum. Following light irradiation, electronic excitation in which electrons jump from valence to conduction band and form positive holes [118]. These holes are transferred among nanoparticles and organic molecules to produce various ROS such as hydroxyl radicals by reacting with oxygen and water molecules; ^1^O_2_ from the reaction between positive holes and oxygen. superoxide radical anions from the reaction of free electrons with oxygen. The ROS acts on microbial pollutants to remove them by photo inactivation [118, 119]

The profound benefits of utilising nanotechnology in water remediation along with the associated mechanisms provide viable water remediation and disinfection techniques when compared to conventional remediation solutions, which lack in providing express efficiency in relation to operating and construction costs as well as time and vast energy consumption [110, 116]. Due to their small size nanomaterials can be fabricated to produce novel and unique physical and chemical properties. Their larger ratio of surface area to volume, unique surface chemistry adjustability and biocompatibility are among the key factors which establish Nanotechnology as a remarkable application in water remediation. Nanotechnology application in wastewater remediation and disinfection provides a solution to the main challenge of recalcitrant microbe removal from different environmental mediums especially wastewaters, and thus better safeguard the ecosystem [108, 117].

## Conclusion

In this review, we highlighted the recent advances related to the photoinactivation of drug-resistant bacteria through aPDI mediated by nanomagnet-porphyrin hybrid coupled systems. As demonstrated here, this emerging research field has generated important results and developments that enable a more efficient treatment of water and wastewater contaminated by drug-resistant microbes. Extensive success of the inactivation and address of DRB within laboratory studies greatly motivates the importance of the need to incorporate aPDI into wastewater treatment systems. The ability of nanomagnet-porphyrin hybrid based aPDI to generate high quantum yields of singlet oxygen species and circumvent the innate defence mechanisms of a broad spectrum of bacteria as well as bypass their capsular shielding mechanisms which is a great limitation among the most recent and current WWTTS. The preferential uptake bias of porphyrin structures by bacterial units especially when immobalised onto nanomaterials affords internal ^1^O_2_ generation, and thus causes complete inactivation of the bacteria from the inside out with no regrowth of the bacteria unlike with existing wastewater treatment methods. One of the factors determining efficient aPDI is the accumulation of a light-activated compound, namely, a PS. Targeted PS recognition is the approach based on the interaction between the membrane receptor on the bacterial surface and the PS, where-as the compound is efficiently accumulated by the same mechanism as the natural ligand as seen with gallium meso-porphyrin IX. Coupled with another mechanism of the antibacterial effect of silver ions related to its affinity for thiol group on bacterial proteins, which interferes with DNA processing. AgNPs have been found to be more potent bactericides than silver ions and other silver salts, possibly due to their extremely large surface area, which provides enhanced reactivity and self-assembly capacity where appropriate. The high inactivation profiles of aPDI, low operating costs and broad-spectrum bacterial application affords aPDI the potential of coming up with the title of being among the most highly effective if not possibly the most effective antimicrobial strategy in wastewater treatment. What remains is for the toxicity of these nanomagnetic and metal hybrid porphyrin systems in water systems to be ascertained and additional, recovery and reuse tests conducted to optimize and increase validity of the sustainable aspect of nanomagnet-porphyrin hybrid centred aPDI. The authors believe that this systematic review can stimulate new researchers and assist in the future development of this important field of research, especially when it comes to the aquatic environment and natural water resources and given the adequate attention this method can aid globally but more so within emerging economies to ensure potable water is delivered to all people. Thus, finally overcoming the issues related to water scarcity and compromised health as previously recalcitrant waterborne disease-causing pathogens associated with health problems will be completely addressed and alleviated.
